# Attitudes, perceived knowledge, and experiences regarding condom use, STIs, transactional sex, and healthcare utilization among young male forced migrants in Stockholm, Sweden: a qualitative study

**DOI:** 10.1080/16549716.2026.2697407

**Published:** 2026-07-09

**Authors:** Jordanos Tewelde McDonald, Benjamin Fayzi, Majdi Laktinah, Anna Mia Ekström, Mariano Salazar

**Affiliations:** a Department of Global Public Health, Karolinska Institutet, Stockholm, Sweden; b Transcultural Centre, Region Stockholm, Stockholm, Sweden; c The Association of Unaccompanied Minors Stockholm, Stockholm, Sweden; d Department of Infectious Diseases/Venhälsan, South General Hospital, Stockholm, Sweden

**Keywords:** Sexual health, sexual behaviors, STIs, condom use, male forced migrants

## Abstract

**Background:**

The experience of forced migration, along with post-migration stressors, can elevate the risk of poor sexual health outcomes among young people. Despite this, little evidence is available on the sexual health needs, behaviors, and healthcare utilization of young male forced migrants in Sweden and elsewhere.

**Objective:**

This study aims to explore the attitudes, perceived knowledge, experiences, and needs of young Eritrean, Afghan, and Syrian men regarding condom use, Sexually Transmitted Infections (STIs), transactional sex, and sexual healthcare utilization.

**Methods:**

In this qualitative study, semi-structured interviews were undertaken with 32 young male forced migrants (former unaccompanied minors, refugees, and asylum seekers) ages 16 to 28. The data were analyzed using qualitative content analysis.

**Results:**

Three broad themes and eight categories emerged from the analysis: ‘Gaps in STI knowledge and ambiguity towards condom use as an STI prevention strategy’; ‘knowing it is illegal to pay for sex in Sweden, yet feeling ambivalent towards transactional sex’; ‘school, female partners, and trust in healthcare providers eased challenges in sexual health communication and access’.

**Conclusions:**

This study highlights important sexual health needs among the study population, emphasizing the value of school-based sexuality education in improving knowledge, including condom use. Female partners also played a key role in facilitating communication, STI testing, and service utilization. To address these needs, school-based interventions should be strengthened to include safer sexual practices, communication skills, and increased awareness of sexual health services. Programs should also provide practical guidance on accessing sexual healthcare and youth-friendly clinics.

## Paper Context

- **Main findings:** Our findings highlight STI knowledge gaps, ambiguous attitudes towards condom use and transactional sex, as well as challenges related to sexual health communication and navigating access to sexual health care and information within our study population.
- **Added knowledge:** Attending school-based sexuality education and having a female partner with sexual health awareness facilitated sexual communication, STI testing, condom use, and the utilization of sexual health care. While various barriers to accessing care in general were identified, those who visited youth clinics perceived the services and staff more positively.
- **Global health impact for policy and action:** It is vital that sexual and reproductive health and rights interventions for young male forced migrants address aspects such as safe sexual practices, effective sexual communication, transactional sex, and navigating sexual health care. Furthermore, these interventions should integrate practical skill-building elements regarding access to care and information to better address the specific needs of this population.

## Background

Unaccompanied refugee minor population has increased globally, peaking in Europe and Sweden in 2015 [1–3]. Most were adolescent boys aged 15–17 years [4–6] with many originating from Afghanistan and Eritrea [2,7]. In 2023, about 43.000 unaccompanied minors applied for asylum in the European Union [5]. Forced migrants, including youth, face elevated risks of poor sexual and reproductive health and rights (SRHR) outcomes both during migration and in host countries [1,4,8–13]. These risks stem from pre-migration factors such as conflict, instability, and limited access to SRH information and care, combined with unsafe journeys, that may involve violence and the loss of social networks. After arrival, challenges such as limited understanding of the new context, discrimination, and psychosocial stressors further increase vulnerability [4,9–11,13].

Moreover, young people with forced migration experiences, including young men and unaccompanied minors, face heightened risks of sexual violence and exploitation [4,11,13], alongside other adverse health outcomes. Altogether, these factors often result in varying levels of health risks and consequences that impact young people’s sexual health [1,2,4,11]. Given the challenges and health risks described above, it is important to recognize that young people with experiences of forced migration constitute a diverse group.

Sexual health is shaped by social and gender norms, which influence young men’s SRH experiences, including those related to intimate relationships [8,14]. For refugee and migrant youth, migrationcan shape the formation of attitudes and understandings of sexual health [15]. Research shows wide variation in SRHR knowledge among refugee and migrant groups, including knowledge of sexually transmitted infections (STIs) [10,16]. Evidence also suggests that men, particularly young men, often have lower levels of sexual health literacy than women [16]. In addition to mixed levels of knowledge and sexual health literacy, sexual risk-taking behaviors also differ within these youth populations. In Sweden, transactional sex (TS) in the form of buying sex is illegal [17]. However, a Swedish study found that migrant youth may be more likely to engage in selling sex the longer they reside in the country, particularly those living alone [12].

In high-income settings such as Sweden, young migrants and refugees often face barriers to accessing and using SRH services [4,18]. These include language difficulties, challenges navigating the healthcare systems, and experiences of discrimination [16,18,19]. Such barriers can affect young men and women differently. Studies from Scandinavia show that young male migrants use SRH services less and more often report negative healthcare experiences than females [18,20]. Evidence also indicates that young men have greater unmet SRH needs compared to both female peers and the broader youth population in Sweden [18,21]. Despite the recognition of these gaps, they are rarely fully addressed in practice [4,22], contributing to ongoing unmet needs and potential negative impacts on young men’s health and well-being [22].

Like many European countries, Sweden experienced a marked influx of young people with forced migration backgrounds in 2015, many of whom were unaccompanied boys and young men from Afghanistan, Syria, and Eritrea [3]. Nonetheless, SRHR research has largely focused on women, with limited attention to young men’s sexual health perceptions, experiences, needs, and behaviors, particularly among those with forced migration backgrounds [4,8,16,20]. Furthermore, there is a lack of knowledge about how these young men experience healthcare services and interact with healthcare professionals [20].

This paper is part of a larger research project in which we explored various topics related to sexual and mental health. We previously reported that the sexual health needs of young Syrian, Eritrean, and Afghan men, focusing on norms, romantic relationships, and sexual consent, changed with time spent in Sweden and were linked to their mental health [23]. In this article, we explore these young men’s attitudes, perceived knowledge, needs, and experiences regarding condom use, STIs, transactional sex, and healthcare utilization, which are all important determinants of sexual health and well-being [8]. Gaining deeper insight into their SRHR perceptions and experiences is essential, as these factors can have lasting effects on health and relationships and help inform more tailored interventions [24,25].

## Theoretical framework – Intersectionality

We applied an intersectional lens to examine how social and contextual factors and identities interact to shape sexual health understanding, needs, behaviors, and experiences of our study population. Intersectionality is an analytical framework for understanding how multiple, socially constructed categories intersect with broader dimensions of inequality in specific contexts [26]. It has been used in public health and migration research to highlight migration-related contextual factors, including pre- and post-migration conditions, and the resulting health inequalities [27]. This approach allows insights into the complexity of the health of various migrant groups and how it is linked to systems of power, privilege, and oppression, leading to different experiences between privileged and marginalized groups [27,28]. The intersectionality approach also underlines that the given meaning of social factors in each setting is individualized and therefore fluid and dynamic [27].

## Methods

### Study setting

This study was conducted in the Stockholm region, which had a population of 2.47 million in 2024, of whom 27.6% (681,538 individuals) were foreign-born [29–31]. Of these, 66.811 were young men aged 15–29 years who were born in Afghanistan, Eritrea, or Syria [29,32]. At the end of 2015, the combined number of young men born in these countries was 33,409 [33] and 16,356 in 2013 [34], representing a 49% increase between 2013 and 2015. In Sweden, individuals with refugee and migrant backgrounds, including youth, may face greater risks of poor sexual health. Accordingly, the national SRHR strategy prioritizes these groups in preventive health efforts [35].

In the Stockholm region, SRH services are provided at both primary and specialized levels in line with the national guidelines. Around 30 youth clinics offer services for those aged 12–22, including SRH information, STI testing and treatment, contraceptive and condom counseling, and psychosocial support [36,37]. Specialized sexual health clinics [38], including the RFSU clinic (The Swedish Association for Sexuality Education) [39] and Stockholm men’s clinic (Mansmottagningen) [40], can be accessed by those aged 18 and older. Among young people, the most common STI is Chlamydia, with 8.862 cases in 2023, 54.9% of which occurred in the 20–29 years age group [41]. Recently, however, gonorrhea cases have increased by 32% compared to 2022 (from 1.863 cases to 2.468 cases) [41]. There is no STI-related data available regarding ethnicity and/or migration status in Stockholm [41].

Like any Swedish resident, asylum seekers and refugee youth under 18 have the right to attend school [42] and to receive subsidized healthcare and information services [43], including youth clinic services and school-based interventions [36,44]. In Sweden, adult asylum seekers are entitled to emergency and dental care, care that ‘cannot be postponed,’ and services under the Communicable Diseases Act, including contraception counseling and care related to maternity and childbirth care [43]. Additionally, both adult and minor asylum seekers have the right to an interpreter and a health examination upon arrival, including assessment of healthcare needs, communicable disease testing, and provision of health information [43].

SRHR information provided to young people includes comprehensive sexuality education (CSE) in schools and multilingual information conveyed via NGOs and various digital platforms (such as youmo.se, UMO.se, rfsu.se) [45–47] among others. In general, there is no standardized educational curriculum related to SRHR for young asylum seekers and refugees who do not attend school [44]. The Transcultural Center, a knowledge center for migration and equitable health and care within Region Stockholm, offers linguistically and culturally adapted sexual and mental health communication for individuals with forced migration backgrounds [48].

### Study design and participants

This exploratory study used an inductive qualitative approach based on semi-structured interviews [49]. This approach enabled in-depth exploration of participants’ attitudes, perceived knowledge, and experiences related to STIs, condom use, healthcare, and transactional sex. Participants were refugee and asylum-seeking young men from Syria, Afghanistan, and Eritrea who met the inclusion criteria: residing in Region Stockholm, aged 15–29, and having arrived in Sweden within the past 10 years. Residence permit status was not an inclusion criterion. This group was selected due to the large number of young men with forced migration experiences arriving in Sweden, including Stockholm, prior to the study [29].

Detailed sampling and recruitment procedures are described in a previous paper from this project [23]. A total of 32 young men participated (15 Afghans, 11 Syrians, and 6 Eritreans), all of whom had sought asylum upon arrival in Sweden, mainly as unaccompanied minors (see Table 1). Most participants reported being single (*n* = 22), although many had previous relationships; the remaining participants were currently in romantic relationships. Two had experienced long-distance relationships during migration that ended after arrival in Sweden. None were married or had children. Overall, 26 participants had engaged in sexual activity, and 11 had used SRH services in Sweden.

**Table 1.** Self-reported characteristics of study participants.

Characteristics	Number of participants (N)
Age (years) 16–20 21–25 26–28 Country of birth Afghanistan Syria Eritrea *Migration status Asylum seekers Refugee status UM with a residence permit Years in Sweden 3–4 5–6 Family situation in Sweden With family/relatives With no family #Educational level High school level University level Sexual identity Heterosexual Bisexual Relationship status In a romantic relationship Single Sexually active (past or present) SRH service utilization (including youth clinics)	17 12 3 15 11 6 3 5 24 18 14 6 26 28 4 31 1 10 22 26 11

*Self-reported migration status at the time of the interviews. UM = formerly unaccompanied minor.

^#^Attending high school or university in Sweden or completed high school in Sweden.

### Data collection

A semi-structured interview guide was developed based on discussions with SRHR professionals and prior literature. The data used in this paper are part of a broader qualitative dataset addressing migration experiences, gender norms, sexual consent, and romantic and sexual relationships, reported elsewhere [23]. The guide also covered topics central to this paper: attitudes toward STI prevention and knowledge, condom use, sexual behaviors (including risk-taking), attitudes toward transactional sex, and access to and use of healthcare services in Stockholm (see Supplementary File 1).

Data were collected between June 2019 and October 2020. The research team covered the three languages represented in the project: Dari, Arabic, and Tigrinya. Interviews in Dari and Arabic were mainly conducted by trained research assistants (second and third co-authors), with the first and last authors co-facilitating initial interviews to ensure consistency and ethical adherence. Clarifications were made through probing questions, with real-time translations provided when needed. After initial supervision, the research assistants conducted interviews independently. Interviews with Eritrean participants were conducted by the first author in Tigrinya. Most interviews were held face-to-face in confidential settings at Karolinska Institutet or at an NGO working with migrant youth. However, four of the interviews in Tigrinya were conducted via Zoom due to COVID-19 related restrictions. The interviews were audio-recorded and lasted between 1 and 2 hours. After each interview, participants completed a short questionnaire consisting of sociodemographic questions.

### Data analysis

Data were analyzed inductively using Graneheim and Lundman’s content analysis (QCA) approach [50]. Recordings were transcribed verbatim by research assistants and the first author, then professionally translated into English. In the initial QCA phase, research assistants and the first author reviewed all audiotapes in their respective languages to verify transcripts and translation quality. Transcripts were then read repeatedly, and meaning units were identified, condensed into codes, and grouped into categories [50].

Initial coding was performed by the first author using Open Code 3.6 software [51]. Next, codes, categories, and themes were refined through discussions with the last author until consensus was reached. Data collection continued until saturation, when no new information emerged. Findings were compared across participant groups, with differences noted when present; overall, similarities predominated. The first author used memo writing throughout the process to capture emerging insights. An intersectionality lens guided interpretation, highlighting how contextual factors and intersecting identities shaped needs, behaviors, experiences, and perceived knowledge.

### Ethics

The Helsinki Declaration [52] was followed, and ethical approval was obtained from the Swedish Ethical Review Authority (Dnr: 2019–01159). Under the Ethical Review Act (Lag (2003:460), §18) [53], individuals aged 15+ may participate in a study without parental consent [53]. Participation was voluntary, and written and verbal informed consent were obtained before each interview. Participants received study information in their mother tongue (written and oral), had opportunities to ask questions, and contacted the research team to consent.

Given the sensitive topics, interviews could cause discomfort. Researchers were trained to provide support and referrals if needed; none were required. All participants received information on health services in the Stockholm region. Data was anonymized, and considerations were given to how we presented our findings, to avoid reinforcing further stigmatization. Participants received two movie tickets.

## Results

The analysis yielded three broad themes, comprising eight categories as shown in Table 2.

**Table 2.** Themes and categories.

Theme	Categories
Gaps in STI knowledge and ambiguity towards condom use as an STI prevention strategy	Awareness of STIs but limited STI knowledge Awareness of effective STI communication with partner for prevention Ambivalent towards condom use
Knowing it’s illegal to pay for sex in Sweden, yet feeling ambivalent toward transactional sex	Ambivalent toward transactional sex and those selling sex Aware that paying for sex is illegal in the Swedish context
School, female partners, and trust in healthcare providers eased challenges in sexual health communication and access	Difficulties communicating sexual health needs and seeking care School and youth clinic facilitates learning about STIs, condoms, and dispelling myths Mixed experiences of using healthcare services

### Gaps in STI knowledge and ambiguity towards condom use as an STI prevention strategy

#### Awareness of STIs but limited STI knowledge

The concept of STIs – including their perceived risks and vulnerability – was mainly brought up in relation to STI transmission routes and the importances of knowing potential romantic and sexual partners’ STI-status prior to engaging in sexual activities. Generally, the participants were aware of STIs (as diseases); however, most struggled to identify specific STIs, mainly mentioning HIV, gonorrhea, and genital warts during the interviews. They revealed that some of their peers were also uninformed about STIs, particularly regarding how STIs are spread, and were unaware of having contracted an STI, failing to recognize signs and symptoms. In few cases, participants described that they neglected or postponed seeking help. This was attributed to feeling overwhelmed in a new country, fear of stigma, and migration-related stress.

I do not go and check if I have it [referring to STIs] … or see a doctor for it (Afghan young man, 19 years)

While some young men revealed that they had contracted an STI themselves after arriving in Sweden, they expressed feeling upset and frustrated upon being diagnosed.

I was very upset after I found out about my illnesses [referring to an STI] and not using condoms, it was very upsetting, and I was very mad at myself (Afghan young man, 21 years)

#### Awareness of effective STI communication with partner for prevention

STI status communication with potential partners was recognized as a crucial practice before having sex. Yet, most expressed feeling ill-equipped for such discussions, which were associated with uncertainty and frustration. The challenges were essentially in initiating and managing such conversations while possibly being perceived as ‘too suspicious of the other person’ or ‘too eager to have sex’. Reflections of other STI prevention practices included getting tested (preferably accompanied by partner) and avoiding engaging in sexual activities with multiple partners. Conversations about STI testing were also perceived as difficult and were often initiated by their female partners, particularly in cross-cultural relationships (with female partners who were Swedish or from countries other than their own).

… with my Swedish girlfriend, in our first sexual encounter … we talked about it, and we went to see a doctor to make sure we did not have any diseases [referring to STIs] … then we had sex without condoms (Afghan young man, 18 years)

#### Ambivalent towards condom use

Awareness of condom use for STI prevention was common among participants, who identified it as the primary STI prevention practice. However, they stated that some of their peers were uneducated about condoms, and discussions of condom use were in many cases prompted by their female partners. The young men particularly believed that condoms ought to be used during sexual activities with a new partner, especially in the case of one-night stands. Within this context, reluctance to use condoms was described as increasing the risk of acquiring a STI.

It is always necessary to use condoms. I think it is important when you do not know each other well … They (condoms) prevent diseases [referring to STIs] (Eritrean young man, 27 years)

Similarly, condoms were considered necessary when there were concerns about STI transmission or when under the influence of alcohol. Participants with a prior STI diagnosis revealed that they consistently used condoms after such an experience and after receiving STI treatment.

I have personal experience of not using them (condoms) … and I was very sick (suffered from an STI) … now I use them (Afghan young man, 20 years)

The participants stated that other benefits of condom use included easier penetration, the ability to engage in prolonged sexual activity, and increased enjoyment for their sexual partner.

The young men described circumstances in which they did not use condoms. For example, they stated that condoms were unnecessary if other contraceptive methods were used and if they had already taken an STI test with negative STI/HIV results (including their partner’s test). At times, condoms were also viewed as a method of birth control. In discussions about contraceptives and condom use, contraception pills were considered a better option than condoms in long-term relationships, alluding to reduced condom use in committed relationships.

When I was in a relationship (with girlfriend), it was not necessary to use condoms. She was on contraceptives … but yes if I was to have sex with a woman I did not know, I would use condoms (Syrian young man, 18 years)

Across participants, the disadvantages mentioned regarding using condoms included reduced enjoyment and sensation during sex.

### Knowing it’s illegal to pay for sex in Sweden, yet feeling ambivalent toward transactional sex

#### Ambivalent toward transactional sex and those selling sex

Views and awareness of transactional sex – both buying and selling – differed among the participants, ranging from condemnation to tolerance of such practices. Those who disapproved, perceived transactional sex as involving deception, manipulation, and pressure by the buyer. They argued that transactional sex exploits and devalues women and diminishes the value of sex. As such, transactional sex was generally considered unsafe.

If women sell their bodies … it devalues the significance of sex and women … it is wrong [referring to selling sex] (Syrian young man, 25 years)

Conversely, the extent to which transactional sex was accepted depended on personal choice regarding participation in such practices, as well as on the condition that mutual consent was granted.

Perceived reasons for selling sex were elaborated on and varied depending on where it occurred, whether in their countries of origin, during transit, or in Sweden. While poverty, as a driver of material need, was the main explanation in their countries of origin, survival, lack of other means, and lack of protection were cited as causes during migration. For instance, in refugee camps in Libya, some young men witnessed young women exchanging sexual favors for water and food to survive. In other countries, such as Iran, Turkey, and Greece, the young men also discussed witnessing sex work as a means of material support and other benefits.

We could see the girls being asked to go to rooms with the guards, and they would have some food and water … I would think that they were lucky … but they told me later … they had to do some things in there [referring to sexual favors] (Eritrean young man, 20 years)

While discussing this issue, one participant disclosed having witnessed a young man being forced to participate in sexual acts to avoid violence on the boat during the migration journey to Europe.

#### Aware that paying for sex is illegal in the Swedish context

In Sweden, a different set of perspectives was shared in relation to transactional sex. Participants recognized that paying for sex is illegal and portrayed sex as difficult to attain. However, involvement in transactional sex, specifically through selling sex, was explained to occur among some young Swedish women who were being exploited due to their need for or addiction to alcohol and/or drugs.

Regarding transactional sex outside of Sweden, participants spoke of a few peers who, for example, traveled to Germany, Turkey, Iran, Greece, or the Netherlands to exchange money for sexual favors. The main reasons for engaging in such practices were to cope with loneliness and relieve mental stress.

Yes, some friends do it, especially in Greece and Turkey as well as in Iran … They [referring to young people] also do it in Sweden, but sometimes girls here sleep with boys because boys provide them with drugs and drinks (Afghan young man, 19 years)

### School, female partners, and trust in healthcare providers eased challenges in sexual health communication and access

#### Difficulties communicating sexual health needs and seeking care

As mentioned earlier, although there was a strong consensus that communication about sex, condom use, and STI with a partner was valuable, it was also described as difficult and often a cause of stress and frustration. Additionally, sharing private sexual information with others, including family members and peers, was described as shameful and could lead to stigmatization, lack of trust, and the spread of personal information as gossip. Stigma, distrust, and shame were also described as barriers to seeking help for sexual health problems, even when they believed or feared they had contracted an STI, which resulted in distress and anxiety.

Yet, trust in well-informed individuals such as close friends, girlfriends, mentors, family members, and healthcare providers facilitated the sharing of private sexual health matters, including concerns about STI-infections, condom use, and seeking needed care.

I would only tell a close friend if I had issues with sex, STIs … and girls … one can also often trust the doctors at the clinics if being worried about having an STI (Eritrean young man, 21 years)

### School and youth clinics facilitate learning about STIs, condoms, and dispelling myths

The young men acknowledged the need, including for their peers, to acquire accurate sexual information since many did receive little or no sexuality education in their country of origin. Access to various sources of information was acquired by attending school-based sexuality education and visiting youth clinics in Sweden. For example, they received information about STIs, masturbation, the negative consequences of unprotected sex, and the benefits of condom use. Additionally, they learned that STIs can spread differently in men and women. One informant stated that he had learned how to put on a condom in ‘the right way’. However, some young men spoke of not having received school-based sexuality education in Sweden.

Discussions about dispelling myths mainly involved the notion that excessive masturbation is harmful. For some participants, masturbation myths and misconceptions were influenced by their religious beliefs, which implied negative health outcomes and social consequences such as backaches, weakened eyesight and physical strength, mental health problems, interest/disinterest in sex, and difficulties in impregnating women. At the time of interview, one participant said:

Masturbation can be harmful … I think when one does it too much, it causes backache and badly affects the eyes. It also reduces physical strength. (Syrian young man,18 years)

### Mixed experiences of using healthcare services

In general, they described the Swedish healthcare system as providing fragmented care. Positive experiences consisted of good reception, positive attitudes from healthcare professionals, and a sense of safety in clinical environments. Despite positive statements, the young men also expressed frustration and dissatisfaction with the system, services, and treatment they received. In the Swedish primary healthcare system (mainly at primary health centers), accessing and receiving care often required long wait times. At times, the services fell short of their expectations. A few examples provided were ineffective treatments, staff lacking cultural understanding, and knowledge about how to treat their condition(s).

The wait to get treatment is so long … and it didn’t help (Syrian young man, 22 years)

It was noted that other newly arrived young men had yet to visit any healthcare facility, apart from the health examination conducted for asylum seekers and newly arrived individuals, and school-based health services. The young men became aware of sexual health services, primarily youth clinics, through female partners, school staff, or personnel at their residential care homes, who supported them in navigating the healthcare system, overcoming language barriers, and accessing care. They visited youth clinics to obtain information on sexuality and relationships, undergo STI testing, and access free condoms, and contraceptive consultation. Those who had visited youth clinics described them very positively compared to other services, emphasizing their focus on youth-centered issues, free services, and friendly staff. The staff at youth clinics were described as competent and professional, providing satisfactory consultations and counselling, as well as being empathetic and responsive.

They treated me very well (healthcare professionals at the youth clinic) … their treatment, work attitudes, and the way they answered questions was all great (Afghan young man, 19 years)

Despite positive experiences at the youth clinics, general knowledge about youth clinics and their services was limited. There was uncertainty about the range of services available at youth clinics and other sexual health-oriented facilities. Among participants who had not yet visited or were unaware of such clinics, a common reflection was that they would utilize youth-friendly services if needed in the future.

I did not know where to go for this kind of care (referring to youth clinics/sexual healthcare services) … now that I know, I would go if I needed such services (Eritrean young man, 20 years)

Trust in the healthcare system, including SRH services and care providers, was shaped by experiences of being understood and respected, as well as by prior positive reception and encounters. These experiences were key factors in participants’ willingness to freely disclose sexual health concerns and seek medical advice. On the other hand, non-adherence to medical treatments and prescriptions, as well as unmet healthcare needs was, as noted earlier, a consequence of cultural barriers and a lack of trust in both the treatments and the proficiency of medical staff.

I think they want to help you [referring to healthcare staff], but you do not know whether the doctor or nurse will actually help you in the way you want and need. It may be a lack of knowledge … it becomes difficult when they do not understand, and their actions do not help. One does not want to go back (Syrian young man, 22 years)

Barriers to accessing care, including SRH care, were shared and included language difficulties, delays in receiving care, and challenges in making medical appointments. Language barriers, in particular, hindered young men from seeking help or care. Another barrier mentioned was exposure to discrimination by certain healthcare providers. Poor knowledge of where and how to access care was also stated. In practice, making appointments digitally or via phone and navigating available services, such as sexual health and STI – testing services, was described as complicated.

For some [referring to peers], they cannot make an appointment … they are afraid because they don’t speak the language well … so they don’t go (referring to healthcare clinics) (Afghan young man, 20 years)

For the young men, other obstacles outside the healthcare system that often-delayed help-seeking, including for SRH services, were related to having no residence permit, experiences of mental health issues, and fear of having an illness/STI.

## Discussion

Our findings show that young male forced migrants in Sweden have gaps in STI knowledge, ambivalent attitudes toward condom use, and difficulties communicating sexual health needs and navigating services. Supportive female partners and school attendance facilitated STI testing, condom use, and dispelled myths about sex and masturbation. Attitudes toward transactional sex were ambivalent and context dependent. Despite reported barriers to care, experiences with youth clinics were generally positive.

Gaps in STI knowledge are consistent with findings from studies in Germany [54] and Sweden [55] among migrant populations. This is concerning, as participants also expressed ambivalent attitudes toward condom use despite recognizing its protective role. Such ambivalence may stem from concerns about sexual performance, reduced pleasure, limited knowledge of correct use, and fear of negative partner reactions [56]. Broader structural barriers, including limited access to healthcare, may further reinforce these attitudes.

Ambivalence toward condom use can lead to inconsistent use, which appears to be common among young people in Sweden, with about 25% reporting condom use during their most recent sexual encounter [57]. Nevertheless, migrants face systemic barriers that may increase the likelihood of non-condom use and the risk of acquiring STIs [58]. For example, a study in Sweden found that young people from various vulnerable groups, including those with migration backgrounds, were overrepresented among STI diagnoses [59]. Without targeted efforts, these knowledge gaps and their consequences are likely to persist and may further worsen health outcomes.

The young men in this study also reported that communicating their sexual health needs was challenging. This may be explained by a lack of trust in the healthcare system, varying levels of individual health literacy, and sociocultural factors that render discussions about sexual matters shameful or inappropriate, as shown in previous research [14].

Participants described how discussions about condom use and STI testing were often initiated by female partners, consistent with studies from Sweden [57,60] and elsewhere [61]. This may reflect limited sexual agency among the participants [62], though it likely applies to young men more broadly rather than being specific to those with migrant backgrounds. For example, Swedish data show that girls often take greater responsibility for sexual communication, while boys tend to have greater needs for knowledge and communication skills regarding sex and relationships [60]. This pattern may stem from the tendency to frame sexual and reproductive health primarily as a women’s responsibility, particularly regarding contraception and pregnancy, thereby overlooking men’s sexual health needs, including those of young male forced migrants [8].

Collectively, these findings highlight key priorities for strengthening comprehensive sexuality education (CSE) in Sweden, including for refugee and asylum-seeking young men, particularly given the decline in condom use among youth in Europe [63]. Moreover, CSE programs targeting youth populations, including young refugees and asylum-seeking youth, should also address norms that support the unequal gendered distribution of responsibility for couples sexual health, as found in our study.

Participants expressed ambivalent attitudes toward transactional sex (TS), ranging from rejection to conditional acceptance. While none reported personal involvement, several were aware of its occurrence within their social circles. Rejection of TS may reflect both personal values and acculturation to norms in Sweden, where the purchase of sexual services is criminalized and gender equality is strongly emphasized, contributing to more negative views of such practices [64].

Engagement in transactional sex is concerning due to its links to increased health risks [65]. Evidence from a Swedish study indicates that, within the past year, the prevalence of TS among young migrants aged 15–25 ranged from 3% to 9% [12]. Notably, a longer duration of residence in Sweden has been associated with a higher likelihood of selling sex. This pattern has been attributed to factors such as acculturation stress and coping-related stress experienced during the integration process [12]. These findings underscore the importance of interventions aimed at improving sexual health among young migrant populations. Such interventions should not only raise awareness of the risks associated with TS but also address the broader structural and socioeconomic determinants that contribute to its occurrence.

A key finding is the important role of schools in improving sexual health knowledge and promoting safer practices, such as condom use. Schools are crucial settings for delivering comprehensive sexuality education programs [66], which can improve outcomes and help bridge knowledge gaps among young people with forced migration backgrounds, as shown in Sweden [67]. This is particularly relevant given that migrant youth under 18 have the right to attend school regardless of migration status [42]. Ensuring equitable access to school-based sexuality education, alongside other SRHR interventions for young adults, is essential. Particular attention should be given to supporting young men in developing the knowledge and skills needed to manage their sexual health and well-being.

Experiences of healthcare use varied but were generally positive, particularly at youth clinics, where responsiveness and empathy appeared to foster trust despite perceived barriers. However, it is important to note that although youth clinics in Sweden are generally well evaluated, users with a migrant background report lower levels of satisfaction than with Swedish-born youth [68]. Notably, many participants attended youth clinics with support from female partners, school staff, or residential care personnel, suggesting a reliance on guidance due to unfamiliarity with the healthcare system and language barriers. As described by Levesque et al. [69], the ability to seek care depends on both autonomy and knowledge of available services. These findings highlight the need to improve awareness of SRH services, including youth clinics, among this population. Although our findings indicate that female partners often facilitate participants’ access to healthcare, their presence during clinical consultations may, in some cases, limit young men’s ability to discuss sensitive topics openly, including violence, coercion, and other personal concerns.

Individual and systemic factors negatively affected participants’ satisfaction with healthcare, including perceived discrimination, language barriers, and limited knowledge of how to navigate the system, findings consistent with previous studies [16,18]. In Sweden, around one in three migrants report experiencing discrimination, with higher rates among young people and men [70]. These findings highlight the need for a better understanding of how structural and individual barriers interact to shape health outcomes and access to care.

### The intersectionality lens

The sexual health needs of the participants largely align with those of young people in high-income settings. However, migration-related experiences intersect with individual factors to shape these needs [1,2,71]. A key intersection involves youth, unaccompanied minor status, and forced migration. Separation from caregivers, disrupted social networks, and potential exposure to violence increase vulnerability and shape specific needs [2]. At the same time, participants are navigating the transition to adulthood, a critical stage in the formation of behaviors and norms [2,71], often without family support and within a new sociocultural context. Consequently, they face overlapping health and adaptation challenges that may limit their access to resources for optimal sexual health.

### Methodological considerations

This study has several strengths. The use of follow-up and probing questions enhanced flexibility, facilitated discussion, and minimized misunderstandings during data collection. Presenting our preliminary findings to researchers not involved in the project (peer debriefing) allowed us to validate our codes, categories, and themes. The multidisciplinary composition of the team (sociology, public health, and medicine) further strengthened the analysis. Selected quotations illustrate that findings were grounded in the data, and a detailed description of the research context supports the transferability of the results.

However, this study had some limitations. Although we used several strategies to enhance the participants’ trust (confidentiality, making sure the participants understood the study aims, etc.), the sensitive nature of some of the topics addressed (e.g. buying/selling sex) may have led participants to withhold or modify some responses. Credibility was strengthened by conducting data collection and analysis concurrently. Some interviews were conducted via Zoom due to the COVID-19 pandemic, which may have affected data quality and interaction; this was addressed through careful probing and clarification. The sample was limited to specific nationalities, which may restrict transferability. While participants varied in age and experiences, the findings are most applicable to young men with forced migration backgrounds in Region Stockholm, particularly those from Afghanistan, Eritrea, and Syria.

### Reflexivity

JTM is a female public health researcher with a migration background, which may have influenced participant interactions and responses. To address this, she engaged in reflexive journaling throughout the study. Her experience in public and sexual health among forced migrant youth, as well as qualitative research in cross-cultural settings, strengthened the study.

ML and BF, young male interviewers who conducted interviews in Arabic and Dari, shared participants’ language, gender, and country of origin, which may have shaped the interview dynamics. MS, together with JTM and AME, contributed global health and cross-cultural research experience, enriching data interpretation. The team reflected on their assumptions regarding sexual health prior to data collection. Shared migration experiences and, in some cases, shared language and origin may have fostered familiarity, while positioning the first author as both an ‘insider’ and ‘outsider’ [72]. These dynamics may have influenced responses and power relations, though their precise impact is difficult to determine.

## Conclusions

This study highlights important sexual health needs among the study population, emphasizing the value of school-based sexuality education in improving knowledge, including condom use. Female partners also played a key role in facilitating communication, STI testing, and service utilization. To address these needs, school-based interventions should be strengthened to include safer sexual practices, communication skills, and increased awareness of sexual health services. Programs should also provide practical guidance on accessing SRHR care and youth-friendly clinics.

## Supplementary Material

Supplementary file 1 Interview guide JTM.docx

## Data Availability

The dataset analyzed in the study can be obtained from the corresponding author upon reasonable request.
