# Supplementary material for: Attitudes, perceived knowledge, and experiences regarding condom use, STIs, transactional sex, and healthcare utilization among young male forced migrants in Stockholm, Sweden: a qualitative study
Source: Glob Health Action. 2026 Jul 9;19(1):2697407. doi: 10.1080/16549716.2026.2697407 (PMC13353457; doi:10.1080/16549716.2026.2697407)
Supplement: Supplementary file 1 Interview guide JTM.docx [file ZGHA_A_2697407_SM6666.docx]

| **Interview guide** |  |
| --- | --- |
| **Topics** | **Questions** |
| **Introductory questions** | I would like to know a little bit about you.  What do you like to do as a hobby? Why?  What does your life look like in Sweden? How so?  Probe: Tell me more |
| **Perceptions of one’s own health** | Now I would like to ask you a few questions about your health.  In general, how would you describe your health?  Probe: How so? What are your thoughts about that? Tell me more |
| **Their journey to Sweden** | Can you describe your experiences of coming to Sweden?  Probes: Tell me more, how did you feel about that? How so? |
| **Sexual experiences, Safe sex, risk-taking, condom use, and transactional sex** | What do you know about condoms?  Some people don't like to use a condom when they have sex. What do you think about that? Why?  Have you ever had sex? Can you tell us about that?  Probe: How did you experience it?  Have you ever used a condom during intercourse? Why? Why not?  Have you had a sexual experience that you regret? Why?  Probe: How did it affect you?  We know that some young people have experiences of having sex for compensation such as a place to sleep, a mobile phone, clothes, work or other things.  Probe: What do you think about that?  Do you know someone who has had sex for compensation? What happened? |
| **Experiences and exposure to sexual coercion** | Do you know someone who has been forced to do something sexual against their will? In what way?  Probe: What do you think about that? |
| **Attitudes, and knowledge of safe sex, STIs, and health care services** | A sexually transmitted infection (STI) is an infection you can get from having sex.  What do you know about STIs?  What can you do to avoid getting/passing on an STI?  What would you do if you were worried about having an STI?  Would you tell anyone? Why? Why not?  **Examine trust, integrity and shame in relation to STIs**  Where do you go if you are worried about having an STI? Why?  Where do you go if you need to talk to someone about STIs? |
| **Attitudes and utilization of health care services in Sweden** | What experiences do you have of Swedish healthcare? Treatments? If none, why?  What services (health services) have you used? (Youth clinic, sexual health clinics, online test or other services) Why?  What do you do if you have questions about relationships, sex and the body? Where do you go? Why?  Explore trust in the healthcare system |
| **Other** | Something you want to add that you think should be included in the interview.  Do you have any questions? |
| **Socio-demographic information** | Age:  Country of birth:  Years in Sweden:  Level of education:  Family situation:  Anything else that you would like to add? |
